# MiR-124 aggravates failing hearts by suppressing CD151-facilitated angiogenesis in heart

**DOI:** 10.18632/oncotarget.24205

**Published:** 2018-01-12

**Authors:** Yanru Zhao, Mengwen Yan, Chen Chen, Wei Gong, Zhongwei Yin, Huaping Li, Jiahui Fan, Xin A. Zhang, Dao Wen Wang, Houjuan Zuo

**Affiliations:** ^1^ Division of Cardiology and Hubei Key Laboratory of Genetics and Molecular Mechanisms of Cardiological Disorders, Tongji Hospital, Tongji Medical College, Huazhong University of Science and Technology, Wuhan, People's Republic of China; ^2^ Department of Cardiology, China-Japan Friendship Hospital, Beijing, China; ^3^ Emergency and Critical Care Center, Beijing Anzhen Hospital, Capital Medical University, Beijing, China; ^4^ Stephenson Cancer Center and Department of Physiology, University of Oklahoma Health Sciences Center, Oklahoma, OK, USA

**Keywords:** miRNA, angiogenesis, hypertrophy, heart failure, CD151

## Abstract

Heart failure (HF) is the final common pathway of various cardiovascular diseases. Although it is well documented that reduction of cardiac angiogenesis contributes to the progression from adaptive cardiac hypertrophy to HF, the molecular mechanisms remain unknown. In the present study, we found that cardiac expression of miR-124 was increased in patients and mice with HF. Recombinant adeno-associated virus (rAAV)-mediated miR-124 over-expression aggravated angiotensin II (Ang II) infusion-induced cardiac dysfunction and abnormal cardiac angiogenesis in mice. *In vitro*, transfection of miR-124 mimics significantly promoted apoptosis and reduced viability, migration, tube formation, and nitric oxide release in endothelial cells. In addition, CD151 was identified as a direct target of miR-124. Endothelial cell injury caused by CD151 silencing was mimicked by miR-124 over-expression. Re-expression of CD151 attenuated miR-124-mediated suppression of cardiac angiogenesis and cardiac dysfunction in Ang II-treated mice. Our observations suggest that miR-124 is an important negative regulator of cardiac angiogenesis and cardiac function, likely by suppressing the expression of CD151 in heart cells. Modulation of miR-124 levels may provide new strategies and targets for HF therapy.

## INTRODUCTION

Heart failure (HF) is a final common consequence of numerous cardiovascular diseases, and a major global healthcare problem [1]. Approximately 5.7 million (2.2%) adults in the United States experienced HF in 2012; the prevalence of HF is expected to increase 46% from 2012 to 2030, resulting in more than 8 million adults with HF [2, 3]. Despite the application of multiple evidence-based therapies, there are numerous patients entering end-stage HF. Thus, an understanding of the mechanisms underlying HF and the development of new intervention strategies are needed.

Cardiac hypertrophy is an adaptive response of the heart against different stressors, but prolonged cardiac hypertrophy leads to HF [4]. Disruption of capillary angiogenesis in the hypertrophied heart plays an important role in the process from adaptive hypertrophy to decompensated HF [5]. During the progression of cardiac hypertrophy, proportional growth of capillaries is required by the increased metabolic and oxygen demands of growing cardiac myocytes. Coronary angiogenesis was enhanced during the initial phase of adaptive cardiac hypertrophy, but reduced as the heart underwent continuous pathological remodeling [6], which could lead to myocardial ischemia and deterioration of hypertrophy.

In addition, less capillary density, indicated by the decreased CD31-positive microvessels, was observed in left ventricular (LV) specimens of patients with worsened LV systolic function [7]. Moreover, coronary microvascular dysfunction, often measured by coronary flow reserve (CFR), is associated with reduced LV ejection fraction, adverse LV remodeling, and decreased long-term survival [8, 9]. Stimulating cardiac angiogenesis to prevent or reverse heart failure may be beneficial [6, 10]. However, the mechanisms underlying the dysregulation of coordinated cardiac angiogenesis remain unclear.

Angiogenesis involves coordinated endothelial cells (ECs) proliferation, migration, branching, and tube formation [11], which can all be regulated by tetraspanins [12]. Tetraspanin CD151, noted for its intense molecular associations with integrins, is abundant on ECs [13]. CD151 coordinates molecular organization of laminin-binding integrins, thereby supporting secondary functions of endothelial cells, which are required for angiogenesis processes [14]. CD151 also maintains endothelial capillary-like structures and the integrity of endothelial cell-cell and cell-matrix adhesions [15]. Deletion of CD151 results in reduced pathologic angiogenesis *in vivo* and *in vitro* [14]. Our early study showed that CD151 gene delivery after myocardial infarction promoted functional neo-vascularization and activated FAK signaling [16]. Recently, it was reported that exogenous CD151 over-expression may promote cardiac angiogenesis and improve cardiac function in rats after acute myocardial infarction [17, 18]. These data suggest that CD151 may be an effective target to regulate cardiac angiogenesis in the transition from adaptive cardiac hypertrophy to HF.

MicroRNAs (miRNAs) are a class of short, conserved, single-stranded and non-coding RNAs that act as negative regulators of target gene expression by suppressing mRNA translation or promoting mRNA degradation [19]. miRNAs are gradually being recognized as important regulators of diverse physiological or pathological processes, including angiogenesis. miR-124 has been reported to significantly suppress angiogenesis and tumor growth in MCF7 cells [20]. Inhibition of mTOR signaling by rapamycin upregulated the expression of miR-124, which was followed by abnormal development of intersegmental vessels of zebrafish embryos [21]. Further, miR-124 was significantly downregulated in glioma specimens and inhibited angiogenesis in glioma [22]. Moreover, the level of miR-124 has been reported to be associated with numerous cardiovascular diseases. Increased miR-124 expression levels were observed in patients with occluded infarct-related arteries in acute coronary syndrome, and may be an indicator for urgent coronary revascularization [23]. A higher level of miR-124 is associated with an increased risk for advanced atherosclerotic disease and subclinical atherosclerosis in smokers [24]. Circulating miR-124 was identified as a prognostic indicator for outcomes after cardiac arrest [25]. However, the role of miR-124 in the regulation between cardiac angiogenesis and HF remains unknown.

In the present study, we found that miR-124 was a key negative regulator of cardiac angiogenesis by targeting CD151. Blockade of miR-124 attenuated abnormalities of cardiac angiogenesis and cardiac dysfunction in Ang II-treated mice. These findings suggest that miR-124 plays an essential role in the transition of adaptive cardiac hypertrophy to HF by impairing cardiac angiogenesis through CD151 inhibition.

## RESULTS

### miR-124 was upregulated in the heart tissues from patients and mice with HF

**Figure 1:**
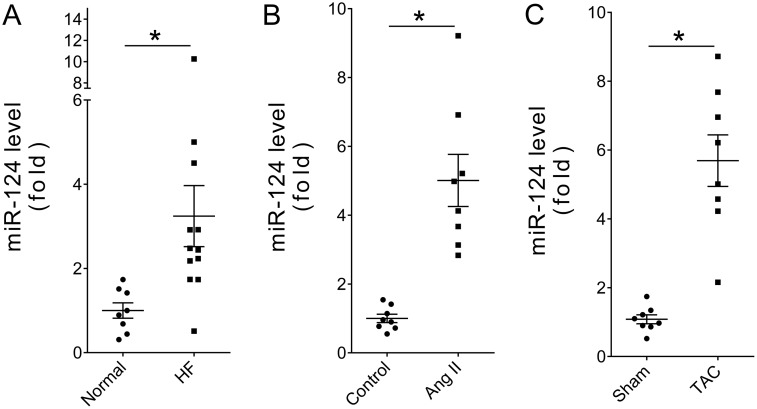
miR-124 was upregulated in failing hearts (**A**) Relative cardiac miR-124 expression in patients with HF as measured by real-time PCR. ^*^P < 0.05 vs. Normal. (**B**) Relative cardiac miR-124 expression in Ang II-treated mice. ^*^P < 0.05 vs. Control. (**C**) Relative cardiac miR-124 expression in TAC-treated mice. ^*^P < 0.05 vs. Sham. *n* > 7. Data are expressed as mean ± SEM.

The expression levels of miR-124 in heart samples from eight traffic accident victims and 12 recipients of heart transplants who suffered with end-stage HF were measured by real-time PCR. The clinical characteristics of the patients have been described previously [26]. The results showed that miR-124 was significantly elevated in human heart samples of HF (Figure 1A). Then, two mouse models of HF induced with angiotensin II (Ang II) infusion and transverse aortic constriction (TAC) were employed. Real-time PCR showed that cardiac miR-124 was consistently increased in both Ang II- and TAC-induced HF (Figure 1B and 1C). These data support the notion that miR-124 may act as a novel regulator in the transition of adaptive cardiac hypertrophy to HF.

### Overexpression of miR-124 aggravated cardiac dysfunction and cardiac microvascular injury induced by Ang II infusion *in vivo*

**Figure 2:**
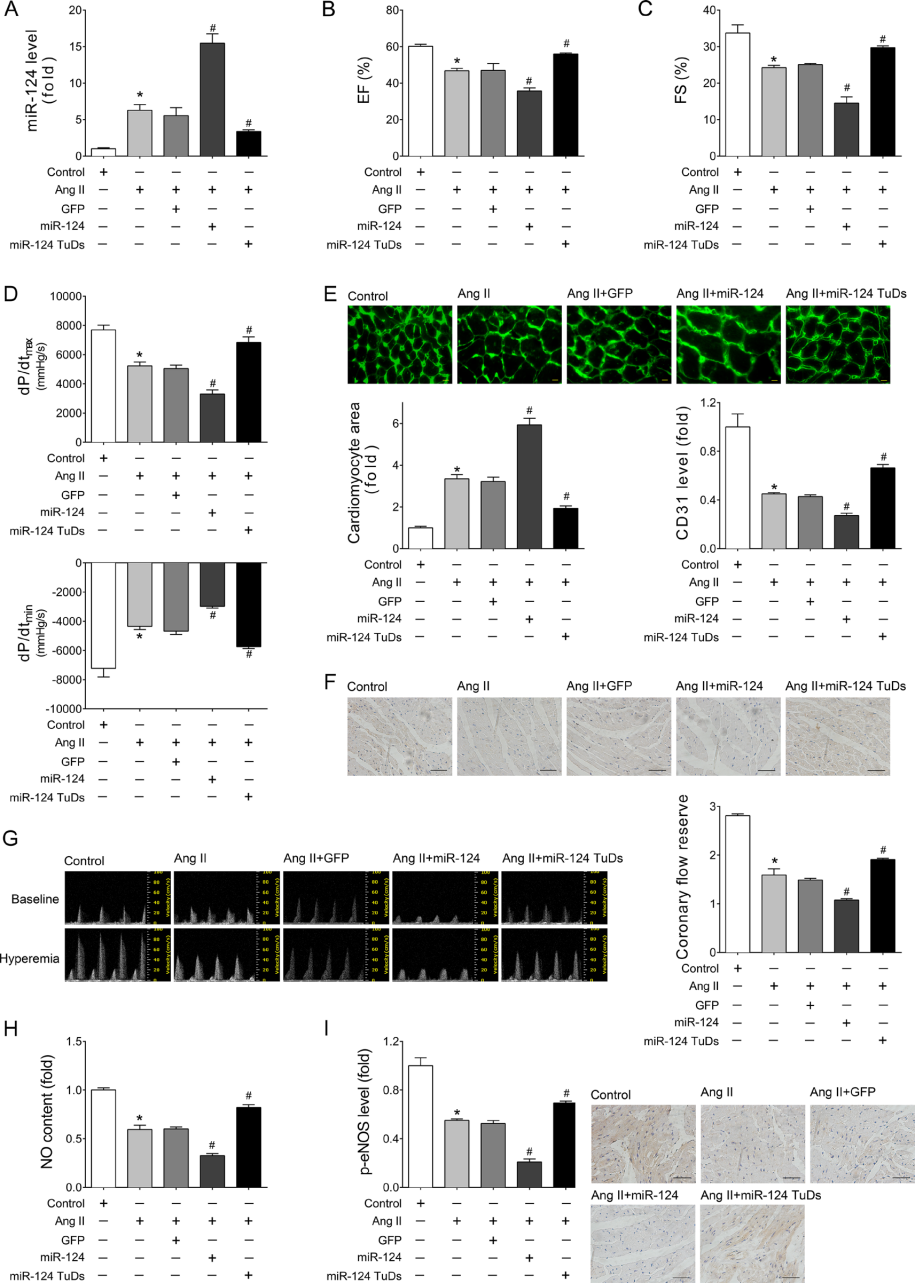
Overexpression of miR-124 aggravated impairment of cardiac function and cardiac angiogenesis induced by Ang II infusion *in vivo* (**A**) Cardiac expression of miR-124 as detected by real-time PCR. (**B–C**) Echocardiographic analyses of mice that received different treatments. (**D**) Hemodynamic detection as measured by Millar cardiac catheter system. (**E**) Representative images of myocardium with WGA staining and quantitative analysis of cardiac myocyte cross-sectional area. Scale bar, 25 mm. (**F**) Representative images of immunohistochemical staining for CD31 in heart tissues. Scale bar, 100 μm. (**G**) Representative images of Pulsed-wave (PW) Doppler of LCA at baseline or under hyperemic conditions induced by inhalation of 1% or 2.5% isoflurane, respectively. CFR is calculated as the ratio of hyperemic peak diastolic flow velocity to baseline peak diastolic flow velocity. (**H**) Cardiac NO content as detected by nitric oxide colorimetric assays. (**I**) Representative images of immunohistochemical staining for p-eNOS in heart tissues. Scale bar, 100 μm. Data are expressed as mean ± SEM, *n* > 7, ^*^P < 0.05 vs. Control, ^#^*P* < 0.05 vs. Ang II.

To explore the effects of miR-124 in cardiac maladaptive hypertrophy and HF, rAAV-miR-124 and rAAV-miR-124 TuDs were used to manipulate the expression of mature miR-124 in Ang II-treated mice. As shown in Figure 2A, rAAV-miR-124 treatment induced miR-124 over-expression in Ang II mice as measured by real-time PCR, while rAAV-miR-124 TuDs delivery decreased the expression of miR-124 (Figure 2A). Echocardiographic and hemodynamic analyses were performed to examine the cardiac function after rAAV and Ang II treatments. The results showed that LV ejection fraction (EF), percentage of fractional shortening (FS), and ±dp/dt were impaired with Ang II-infusion (Figure 2B–2D). rAAV-miR-124 treatment further exacerbated the cardiac dysfunction, while downregulation of miR-124 by rAAV-miR-124 TuDs alleviated the impairment (Figure 2B–2D). Consistently, overexpression of miR-124 aggravated the increase in cardiomyocyte size induced by Ang II infusion, while rAAV-miR-124 TuDs treatment reduced the development of cardiac hypertrophy (Figure 2E).

Two major features of cardiac angiogenesis, microvascular density and function, were investigated. CD31 immunohistochemical assays showed that rAAV-miR-124 treatment further decreased the microvascular density in Ang II-treated mouse hearts (Figure 2F). In contrast, these declines were reversed in the rAAV-miR-124 TuDs group (Figure 2F). Coronary flow reserve (CFR), ECs-derived NO, and the level of activated endothelial isoform of NO synthase (eNOS) were detected to assess the function of cardiac microvessels. Compared with the Ang II infusion group, additional over-expression of miR-124 resulted in a dramatic decrease in CFR, while downregulation of miR-124 attenuated the coronary microvascular dysfunction (Figure 2G). Ang II infusion also induced reduction of cardiac nitric oxide (NO) content and eNOS phosphorylation was aggravated by miR-124, while miR-124 inhibition reversed the effects (Figure 2H and 2I). These findings suggest that over-expression of miR-124 exacerbates the reduction in microvascular density and endothelial injury, as well as cardiac dysfunction induced by Ang II infusion.

### Overexpression of miR-124 impaired endothelial cell angiogenesis *in vitro*

**Figure 3:**
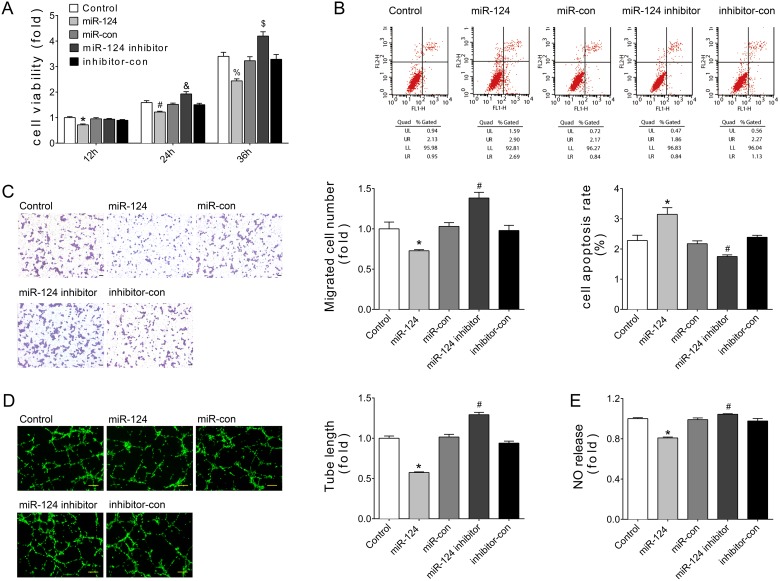
miR-124 reduced angiogenesis of HUVECs *in vitro* (**A**) Cell viability measured by CCK8 kit at 12, 24, or 36 hours after transfection. ^*^P < 0.05 vs. miR-con-12h, ^#^*P* < 0.05 vs. miR-con-24h, ^&^*P* < 0.05 vs. inhibitor-con-24h, ^%^*P* < 0.05 vs. miR-con-36h, ^$^*P* < 0.05 vs. inhibitor-con-36h. (**B**) Apoptosis as detected by Annexin V/PI flow cytometry analysis. (**C**) Cell migration as evaluated by transwell assays. Scale bar, 200 μm. (**D**) Tube formation determined on Matrigel. Scale bar, 200 μm. (**E**) NO release detected by nitric oxide colorimetric assays. Data are representative of three experiments. Data are expressed as mean ± SEM, *n* ≥ 3, ^*^P < 0.05 vs. miR-con, ^#^*P* < 0.05 vs. inhibitor-con.

Endothelial cells’ (ECs) behaviors are the key events in angiogenesis and vascular function. To investigate the role of miR-124 in cultured ECs, gain/loss-of-function analyses were conducted by transfection of miR-124 mimics or inhibitor. CCK-8 assays indicated that over-expression of miR-124 inhibited the viability of HUVECs in a time-dependent manner, while miR-124 inhibitor resulted in opposite effects (Figure 3A). Meanwhile, Annexin V/PI staining assays showed that transfection of miR-124 mimics increased the apoptotic cell proportion (Figure 3B). Transwell experiments, tube formation assays, and NO release detection were employed to detect the functions of ECs. Consistently, HUVEC with miR-124 transfection exhibited impaired cell migration, tube formation, and NO release (Figure 3C–3E). Conversely, miR-124 inhibitor improved ECs’ ability to migrate, form tubes, and release NO (Figure 3C–3E). Together, these data demonstrate the anti-angiogenic effects of miR-124.

### CD151 is a target of miR-124

**Figure 4:**
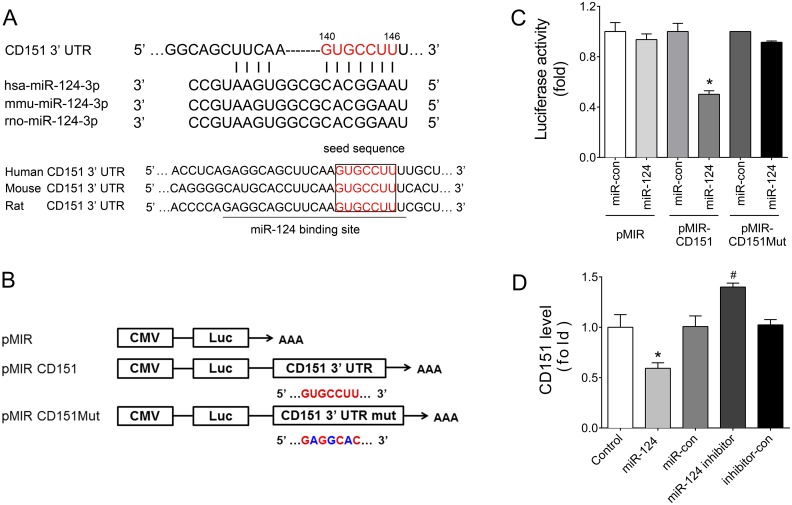
CD151 was a target of miR-124 (**A**) Sequence alignment between miR-124 and the 3′ UTR of CD151 among several species. (**B**) Schematic diagram of the luciferase reporter plasmids of pMIR-CD151 and pMIR-CD151Mut, and the potential target site of miR-124 on the 3’ UTR of CD151. (**C**) Regulation of miR-124 on 3′ UTR of CD151 in HEK293 cells as shown by luciferase reporter assays. ^*^P < 0.05 vs. pMIR-CD151 + miR-con. (**D**) CD151 protein levels of HUVECs with different treatments detected by flow cytometry analysis. ^*^P < 0.05 vs. miR-con, ^#^*P* < 0.05 vs. inhibitor-con. Data are representative of three experiments. Data are expressed as mean ± SEM, *n* ≥ 3.

Using miRNA target prediction programs, we found that CD151 was a putative miR-124 targets, and the predicted binding sites were highly conserved during evolution (Figure 4A). To validate this finding, dual luciferase assays were performed. The 3’ UTR of human CD151 gene (wild-type or seed region mutated sequence) was cloned to pMIR-report vector, named pMIR-CD151 or pMIR-CD151Mut, respectively (Figure 4B). The results showed that after co-transfection with miR-124 mimics into HEK293 cells, the relative luciferase activity of pMIR-CD151 reporter was significantly reduced compared with miR-con (Figure 4C). However, this suppressive effect of miR-124 was abolished by mutating CD151 3’ UTR (Figure 4C). Further, flow cytometry assays showed that miR-124 mimics transfection significantly reduced CD151 expression, and miR-124 inhibitor increased CD151 expression in HUVECs (Figure 4D). These results suggest that miR-124 inhibits CD151 expression by directly binding to its 3′ UTR.

### Down-regulation of CD151 impaired endothelial cell angiogenesis *in vitro*

**Figure 5:**
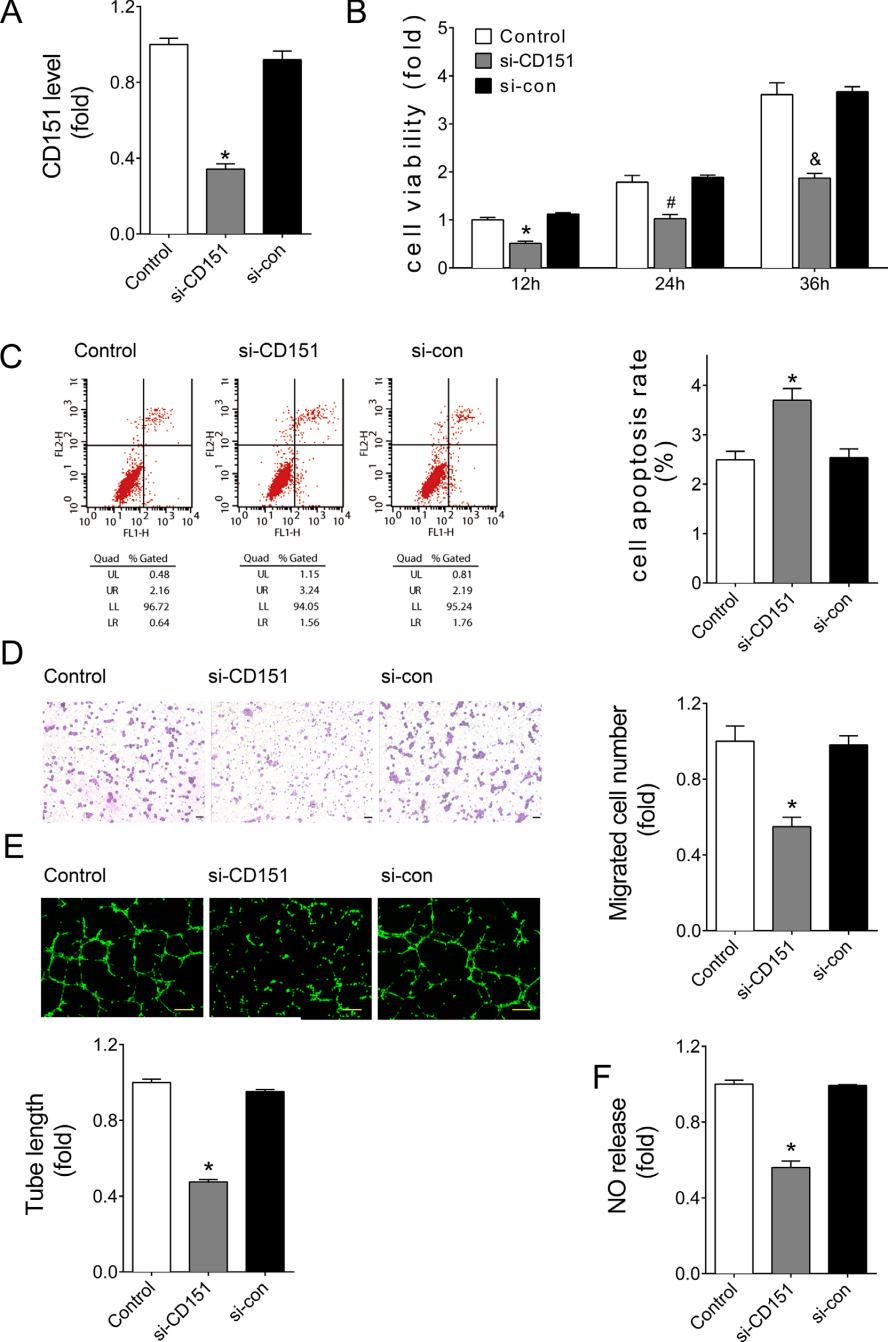
Downregulation of CD151 inhibited angiogenesis of HUVECs (**A**) CD151 protein levels of HUVECs with siRNA treatments as detected by flow cytometry analysis. (**B**) Cell viability measured by CCK8 kit at 12, 24, or 36 hours after transfection. ^*^P < 0.05 vs. si-con-12h, ^#^*P* < 0.05 vs. si-con-24h, ^&^*P* < 0.05 vs. si-con-36h. (**C**) Apoptosis detected by Annexin V/PI flow cytometry analysis. (**D**) Cell migration evaluated by transwell assays. Scale bar, 200 μm. (**E**) Tube formation determined on Matrigel. Scale bar, 200 μm. (**F**) NO release detected by nitric oxide colorimetric assays. Data are representative of three experiments. Data are expressed as mean ± SEM, *n* ≥ 3, ^*^P < 0.05 vs. si-con.

To verify the function of CD151 in ECs behaviors, siRNA against CD151 was transfected into HUVEC. The knockdown efficiency of the siRNA was almost 70% at the protein level detected by flow cytometry (Figure 5A). Consistent with the effects of miR-124, results showed that downregulation of CD151 by siRNA significantly reduced the viability of HUVECs (Figure 5B) and promoted cell apoptosis (Figure 5C). Similarly, knockdown of CD151 induced endothelial dysfunction, as demonstrated by reduced migration (Figure 5D), impaired tube formation (Figure 5E), and damaged NO release (Figure 5F).

### Restored CD151 eliminated the miR-124-induced cardiac dysfunction and cardiac microvascular injury in Ang II-treated mice

**Figure 6:**
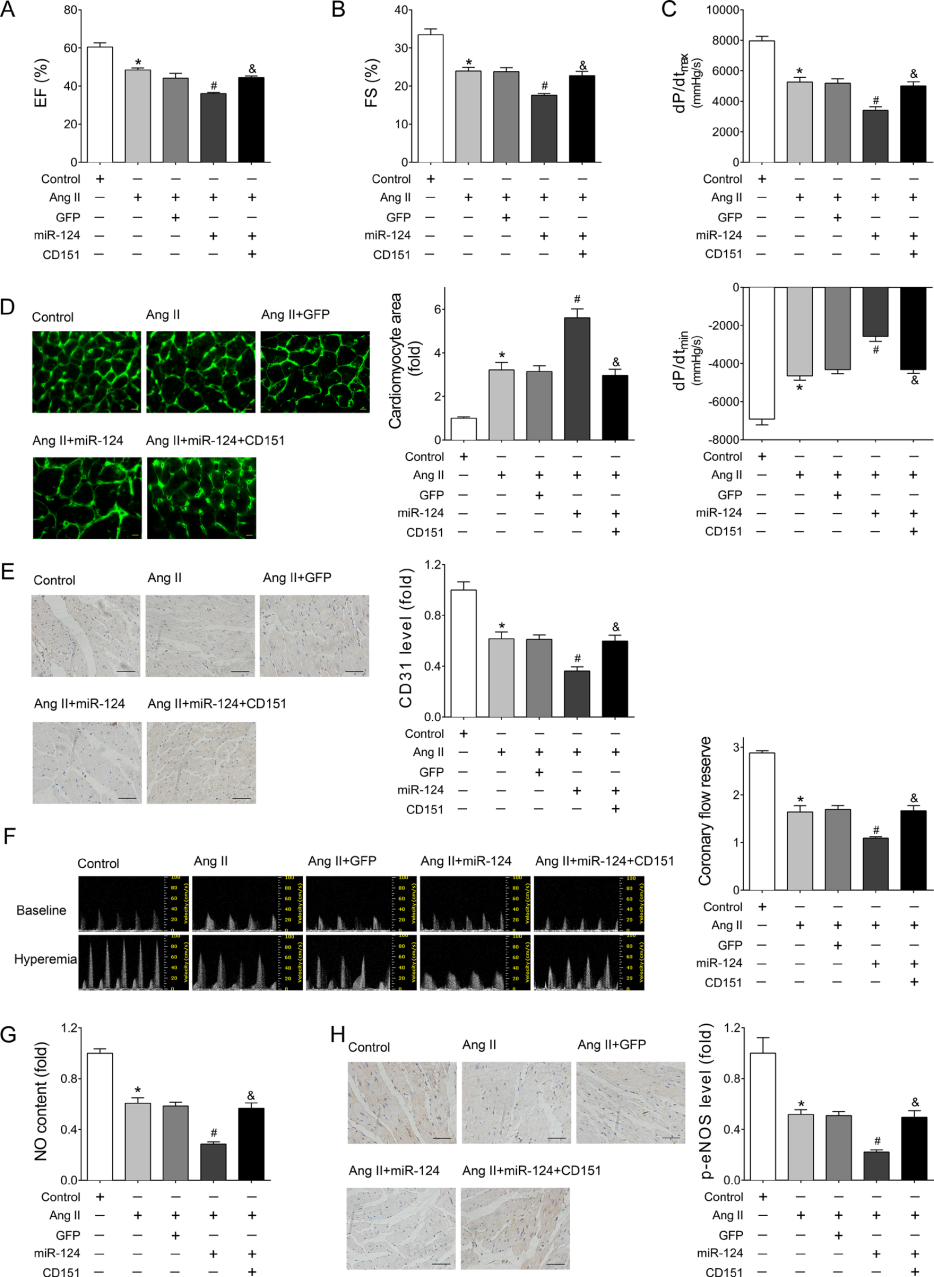
Re-expressed CD151 eliminated the miR-124-induced cardiac dysfunction and cardiac microvascular injury in Ang II-treated mice (**A–B**) Echocardiographic analyses of mice that received different treatments. (**C**) Hemodynamic detection of mice that received different treatments as measured by Millar cardiac catheter system. (**D**) Representative images of myocardium with WGA staining and quantitative analysis of cardiac myocyte cross-sectional area. Scale bar, 25 mm. (**E**) Representative images of immunohistochemical staining for CD31 in heart tissues. Scale bar, 100 μm. (**F**) Representative images of PW Doppler of LCA at baseline or under hyperemic conditions and calculated CFR. (**G**) Cardiac NO content detected by nitric oxide colorimetric assays. (**H**) Representative images of immunohistochemical staining for p-eNOS in heart tissues. Scale bar, 100 μm. Data are expressed as mean ± SEM, *n* > 7, ^*^P < 0.05 vs. Control, ^#^*P* < 0.05 vs. Ang II, ^&^*P* < 0.05 vs. Ang II + rAAV-miR-124.

To verify the role of the miR-124/CD151 pathway in HF, we re-expressed CD151 in rAAV-miR-124-treated mice using rAAV-CD151, which contained the coding sequence of human CD151 gene. The results showed that restored CD151 expression markedly eliminated the destructive effects of miR-124 over-expression in Ang II-induced cardiac dysfunction as determined by EF (Figure 6A), FS (Figure 6B), and ±dp/dt (Figure 6C). In addition, restored CD151 alleviated the rAAV-miR-124-induced increased cardiomyocyte size in Ang II-infused mice (Figure 6D). Regarding Ang II-induced cardiac microvascular injury, enforced CD151 expression counteracted the deleterious effects of rAAV-miR-124, as indicated by increased CD31 expression (Figure 6E), restored CFR (Figure 6F), and enhanced levels of NO and p-eNOS in the heart tissues (Figure 6G and 6H).

## DISCUSSION

In the present study, we identified miR-124-mediated impairment of cardiac angiogenesis by targeting CD151 in HF. Our data showed that cardiac expression of miR-124 was increased in patients with HF as well as Ang II- and TAC-treated mice. Over-expression of miR-124 aggravated Ang II-induced cardiac dysfunction and abnormalities of cardiac angiogenesis in mice, while knockdown of miR-124 protected mouse hearts from the impairments induced by Ang II infusion. Transfection of miR-124 mimics promoted HUVECs apoptosis and reduced HUVECs viability, migration, tube formation, and NO release *in vitro*. Furthermore, CD151 was predicted and verified as a target of miR-124 in ECs. Re-expression of CD151 abolished the damaging effects of miR-124 in HF. Taken together, these results suggest that miR-124 suppresses cardiac angiogenesis by directly targeting CD151 in HF.

Cardiac hypertrophy is an adaptive response to overload, but the growth of cardiac capillaries does not always keep pace with the increased metabolic and oxygen demands of the hypertrophic cardiac myocytes [27, 28]. The disproportional growth between capillaries and myocytes may cause myocardial hypoxia and contribute to pathological cardiac hypertrophy. Data suggest that impaired cardiac angiogenesis is crucially involved in the transition from cardiac hypertrophy to HF [5, 29, 30].

In the present study, we observed the impairment of cardiac angiogenesis in hearts under prolonged pressure overload. Cardiac microvascular density and function were major features of cardiac angiogenesis. CD31 staining showed a loss of microvascular density, while cardiac microvascular dysfunction was demonstrated by decreased CFR and cardiac NO content in the failing hearts. However, the mechanisms of dysregulation of coordinated cardiac angiogenesis were far from clear.

Here, we present substantial evidence supporting the anti-angiogenesis effects of miR-124 in the hypertrophic heart. Over-expression of miR-124 by delivery vector aggravated the loss of cardiac microvessel density and function, which led to subsequent cardiac dysfunction in Ang II-treated mice. Inhibition of endogenous miR-124 expression alleviated the abnormalities of cardiac angiogenesis in the hypertrophic heart, and thereby impeded HF. Our observations support the idea that the aberrant expression of miR-124 contributed to the process from cardiac hypertrophy to HF by impairing angiogenesis.

It was reported previously that miR-124 aggravated the hypertrophic response of cardiomyocytes to Ang II, probably by increasing endoplasmic reticulum stress *in vitro* [31]. miR-124 was reported to inhibit the transdifferentiation from bone marrow-derived mesenchymal stem cells into cardiomyocytes in heart repair after injury by targeting STAT3 [32]. Thus, the aggravated role of miR-124 in failing hearts might also comprise the direct effects of miR-124 on cardiac myocytes. Recently, some researchers focused on the effects of miR-124 in different vascular cell types. miR-124 was identified as a critical regulator of vascular smooth muscle cell (VSMC) function and behavior in neo-intima hyperplasia, and inhibition of miR-124 significantly increased proliferation and migration of VSMC [33, 34]. Decreased expression of miR-124 contributed to an activated phenotype of hypertensive pulmonary adventitial fibroblasts with advanced proliferation, migration, and pro-inflammatory activation [35]. However, few reports have examined the role of miR-124 in ECs’ function.

miR-124 is one of the five most abundant miRNAs (miR-99a-5p, miR-128, miR-124, miR-22-3p, and miR-99b-5p) embedded in the human circulating vesicles [36], which suggests a high priority in the regulation of ECs’ behaviors. In the current study, we found for the first time that over-expressed miR-124 inhibited cultured ECs’ viability, promoted apoptosis, and impaired migration, tube formation, and NO release.

Moreover, we verified CD151 as a direct target of miR-124. CD151, a tetraspanin protein family member, is associated tightly with integrins (α3β1, α6β1, α6β4 and α7β1), and thereby modulates integrin-dependent cell morphology, migration, signaling, and adhesion strength [37]. Although no obvious blood vessel deficiencies were observed in CD151-deleted mice or humans with CD151 mutations [38–40], CD151-deleted mice showed impaired pathologic angiogenesis in different models [14]. The protective effects of CD151 in rats suffering acute myocardial infarction have been reported [17, 18].

In the present study, CD151 silencing by siRNA transfection disrupted cultured ECs’ function, viability, and apoptosis control, consistent with the effects of miR-124 transfection. Furthermore, re-expression of CD151 alleviated miR-124-induced cardiac dysfunction in Ang II-treated mice. Regarding cardiac angiogenesis, re-expression of CD151 weakened the deleterious effects of miR-124, as indicated by restored cardiac CD31, p-eNOS, and NO levels, as well as improved CFR. Therefore, our data suggest that the miR-124/CD151 pathway may suppress cardiac angiogenesis in HF.

It was reported that CD151-null mouse lung endothelial cells were relevant to functional damage and signaling defects, particularly the diminished activation of PKB/c-Akt, eNOS, Rac, and Cdc42 [14]. Our previous study showed that CD151 promoted angiogenesis in bovine aortic endothelial cells and activated the FAK, ERK, PI3K/Akt/eNOS, and Rac1/Cdc42 signaling pathways [41]. Consistently, we found that over-expressed miR-124 significantly decreased the eNOS phosphorylation and NO content in the heart, while re-expressed CD151 restored both.

In contrast, downregulation of miR-124 attenuated the loss of p-eNOS and NO levels in the transition from cardiac hypertrophy to HF, and thereby improved cardiac function. ECs-derived NO, mostly produced by the activated endothelial isoform of NO synthase (eNOS), is an important determinant of endothelial and cardiac function [42, 43]. Interestingly, the cardioprotective effects of corticosteroids [44], insulin [45], and VEGF [46] were reported to arise from the increased vascular NO production following eNOS phosphorylation at the serine residue 1177 (Ser-1177; human sequence) via the PI3K/Akt-signaling pathway. In the present study, the destructive effects of miR-124/CD151 on cardiac angiogenesis and HF may be due to the attenuated activation of eNOS phosphorylation and NO production. However, other possible effects of miR124/CD151 on other pro-angiogenic and anti-angiogenic signaling pathways deserve further investigation.

Here, the rAAV system was applied to manipulate the expression of miR-124 and CD151 *in vivo*. As an extraordinarily promising gene therapy delivery system, the rAAVs present numerous advantages, such as little immunogenicity, sustained transgene expression, availability of different serotypes, and application via the intravenous delivery route [47]. Furthermore, the clinical benefits and safety of gene therapy using rAAVs were reported for the treatment of human diseases, such as Leber's congenital amaurosis [48, 49]. Moreover, we detected the liver and renal function and morphologic features of mice that received rAAVs injections. As shown in Supplementary Figure 1, there were no significant differences among groups that received rAAVs injections, suggesting an excellent safety profile for gene delivery via rAAVs. These findings suggest a possible application for our rAAVs to manipulate the expression of miR-124 *in vivo*.

Our study suggests that miR-124 plays a suppressive role in angiogenesis and endothelial function via CD151, which contributes to the transition of adaptive cardiac hypertrophy to HF. These findings put forward a potential target for promising therapeutic intervention in HF.

## MATERIALS AND METHODS

### Reagents

RPMI-1640, Dulbecco's Modified Eagle Medium (DMEM), and fetal bovine serum (FBS) were obtained from GIBCO (Grand Island, NY). Lipofectamine 2000 (Lipo 2000) reagent was obtained from Invitrogen (Life Technologies Corporation, Carlsbad, CA). The primers for miR-124 and U6 real-time PCR, miR-124 mimics, miR-124 inhibitor, CD151 siRNA, and their controls were purchased from RiboBio (Guangzhou, China). Antibody against CD151 (Cat No: ab33315) was procured from Abcam (Cambridge, MA). Antibody against Phospho-NOS3-S1177 (Cat No: AP0421) was purchased from ABclonal Biotech (Cambridge, MA). Prestained protein markers were obtained from Fermentas (Thermo Fisher Scientific Inc., Rockford, IL). Polyvinylidene difluoride (PVDF) membranes were from Millipore (Merck KGaA, Darmstadt, Germany). Horseradish peroxidase-conjugated secondary antibodies and enhanced chemiluminescence reagents were from Pierce Biotechnology (Thermo Fisher Scientific Inc., Rockford, IL). Alexa Fluor^®^ 594 Donkey Anti-Mouse IgG (H+L) Antibody (Cat No: A-21203) were obtained from MOLECULAR PROBES (Thermo Fisher Scientific Inc., Rockford, IL). Other reagents were purchased from the Sigma-Aldrich Company, unless otherwise specified.

### Human heart samples

The study was approved by the Ethics Review Board of Tongji Hospital, Tongji Medical College, and conformed to the principles of the Declaration of Helsinki. Human heart samples were collected at Tongji Hospital (Wuhan, China). Informed consent was signed by the subjects participating in the study or by their immediate family members in cases of incapacity.

### Preparation and construction of recombinant adeno-associated virus (rAAV)

To manipulate the expression of miR-124 *in vivo*, the rAAV (type 9) was used. The rAAV system (type 9) was a kind gift from Dr. Xiao (University of North Carolina Eshelman School of Pharmacy, Chapel Hill, NC) [50]. For the overexpression of miR-124, oligonucleotides were designed as miR-124 (5′- GATCCGGCATTCACCGC GTGCCTTATTCAAGAGATAAGG CACGCGGTGAATGCCCCGC-3′) according to the mature sequence of hsa-miR-124-3p provided by miRBase (Accession: MIMAT0000422). To achieve the efficient and long-term-suppression of miR-124, tough decoy RNAs (TuDs) were employed as described previously [51, 52]. The oligonucleotides were designed as miR-124 TuDs (5′- GATCCGACGGCGCTAGGATCATCAA CGGCATTCACCATCTGCGTGCCTTACAAGTATTCT GGTCAACAGAATACAACGGCATTCACCATCTGC GTGCCTTACAAGATGATCCTAGCGCCGTCTTCCG C-3′). The oligonucleotides and their reverse complements were synthesized by BGI Tech (Shenzhen, China) and were then then annealed and ligated into rAAV vectors. For the expression of CD151, the full-length sequence of its protein coding sequence (CDS) was amplified by PCR using the primers and then ligated into rAAV vectors. The rAAVs were packaged by triple-plasmid co-transfection in HEK293 cells and were purified as described previously [53]. The resultant rAAVs were designated as rAAV-miR-124, rAAV-miR-124 TuDs, and rAAV-CD151, respectively.

### Animal treatment and gene delivery

All animal experiments were approved by the Institutional Animal Research Committee of Tongji Medical College and complied with the Guide for the Care and Use of Laboratory Animals published by the United States National Institutes of Health. Male C57BL/6 mice (22–25 g) were obtained from the Model Animal Research Center of Nanjing University (Nanjing, China). Mice were housed at the animal care facility of Tongji Medical College with 12-hour light/12-hour dark cycles and free access to water and food. Mice were subjected to thoracic aorta constriction (TAC) or the same operation without aortic constriction for 4 weeks, as described previously [54]. C57BL/6 mice were continuously infused with Ang II (1.5 mg/kg/day, Sigma-Aldrich China Inc., Shanghai, China) with implanted mini-osmotic pumps (Alzet model 1004; DURECT Corp., Cupertino, CA) over a period of 28 days. C57BL/6 mice were randomly divided into different groups (*n* > 7 per group), as follows: Control, Ang II, Ang II + rAAV-GFP, Ang II + rAAV-miR-124, Ang II + rAAV-miR-124 TuDs, and Ang II + rAAV-miR-124 + rAAV-CD151.

For gene delivery, mice were treated with intravenous injection of the corresponding rAAV (1 × 10^11^ virions particles in 100 uL of saline solution) via tail vein. Two weeks later, pressure overload induced by Ang II infusion was performed as described above. Vehicle (0.9% normal saline) was used in control mice. Four weeks later, all of the mice were euthanized and tissue samples were collected for paraffin embedding or freezing in liquid nitrogen, followed by storage at −80°C. Mice were anesthetized with intraperitoneal injection of xylazine (5 mg/kg) and ketamine (80 mg/kg) mixture before they were euthanized.

### Echocardiography

Echocardiography analysis was performed using a Visualsonic Vero 770 System echocardiograph (VisualSonics, Toronto, Canada) with a 30-MHz high-frequency scanhead, as described previously [26]. The measurement of coronary flow reserve (CFR) was performed as described previously [55].

### *In vivo* hemodynamics

After mice were anesthetized, hemodynamic analyses were performed using a pressure-volume catheter (Millar 1.4F, SPR835, Millar Instruments, Inc. Houston, TX) as described previously [56].

### RNA extraction and detection

RNA was isolated from frozen heart tissues with TRIzol Reagent (Invitrogen, Carlsbad, CA) according to the manufacturer's protocol. Total RNA (1 ug) was reverse transcribed using a first-strand cDNA synthesis kit (Thermo Fisher Scientific, Inc., Rockford, IL). The primers of miR-124 or U6 small nuclear RNA and Maxima SYBR Green/ROX qPCR Master Mix (Thermo Fisher Scientific Inc., Rockford, IL) were used for real-time PCR to detect the relative expression of miR-124 with the 7900HT Fast RealTime PCR system (Applied Biosystems, Foster City, CA) according to the manufacturer's protocol. U6 small nuclear RNA was used as endogenous control to miR-124.

### Histology and immunohistochemical staining

Formalin-fixed heart tissues were paraffin-embedded, cut into 4-mm-thick sections, and stained with FITC-conjugated wheat germ agglutinin, CD31, or phosphorylated eNOS (p-eNOs), according to the method described previously [57, 58]. Images were acquired by light microscope and measured by IMAGE PRO-PLUS Software 6.0 (Media Cybernetics, Bethesda, MD).

### Cell culture and transfection

Human umbilical vein endothelial cells (HUVECs) and HEK293 cells were obtained from American Type Tissue Collection and cultured in RPMI-1640 or DMEM supplemented with 10% FBS, respectively. Cells were grown in a humidified atmosphere of 95% air and 5% CO_2_ at 37°C. Cells were transfected with miR-124 mimics (100 nM, similarly hereafter), miR-124 inhibitor (100 nM), siRNA against human CD151 (100 nM), or their negative control (100 nM), using Lipo 2000 reagent according the manufacturer's protocol.

### Cell viability

HUVECs were cultured in 96-well plates and transfected. After 12, 24, or 36 hours of transfection, cells were incubated with 10% Cell Counting Kit-8 (CCK-8) reagent (100 ul) for 1 hour at 37°C. Cells were then measured at 450 nm with a BioRad ELISA reader (Richmond, CA).

### Flow cytometry

Forty-eight hours after treatments with miRNAs as described above, cell apoptosis was determined using an Annexin V-FITC Apoptosis Detection Kit (BD, San Jose, CA) as described previously [59].

For the measurement of CD151 expression, cells were harvested and resuspended in ice-cold 3% BSA/PBS. Approximately 5 × 10^5^ cells in a volume of 100 ul were incubated with CD151 antibody (dilution 1:100) for 30 min at 4°C. Then, cells were washed three times and incubated with Alexa Fluor^®^ secondary antibody (dilution 1:500) in 3% BSA/PBS for 30 min at 4°C in the dark. Finally, cells were washed three times and analyzed on the FACStar-Plus flow cytometer (BD, Franklin Lakes, NJ).

### Migration

Forty-eight hours after transfection, HUVECs (2 × 10^5^ cells per well) were implanted into transwell inserts with 8 μm-pore size membranes (Corning Life Sciences, Corning, NY). After incubating for 12 hours, stationary cells were wiped and removed from the upper surface of the transwell insert membranes. Then, upper chambers were fixed and stained with crystal violet. The migrated cells were counted in five random fields under microscopy (100×, Nikon).

### Tube formation

HUVECs (2 × 10^4^ cells per well) were plated in a 96-well plate pre-coated with 100 μl Matrigel (Corning Life Sciences, Corning, NY) 48 h after transfection. Each well was stained with Calcein, AM (Cat No: C3099, Thermo Fisher Scientific Inc., Rockford, IL) following the manufacturer's protocol after seeding for 6 h. Images were taken with an inverted microscope (40×, Nikon).

### Nitric oxide detection

The content of nitric oxide (NO) in heart samples or cultured supernatants 48 hours after transfection was determined using a Nitric Oxide Colorimetric Assay Kit (Biovision, Mountain View, CA) according to the manufacturer's instructions.

### Target prediction of miRNA

TargetScan (http://www.targetscan.org/) and RNAhybrid (http://bibiserv.techfak.uni-bielefeld.de/rnahybrid/) bioinformatic prediction websites were applied to predict the targets of miR-124.

### Dual luciferase assay

For dual luciferase assay, 400 ng of pMIR-CD151 3′ UTR, pMIR-CD151 3′ UTR mutant, or the empty vector was transfected into HEK293 cells with 40 ng of pRL-TK plasmid (Promega, Madison, WI), respectively. At the same time, miR-124 mimics or miR-con were co-transfected with those reporter plasmids at a final concentration of 100 nM. Luciferase activity was detected as described previously [58].

### Statistical analysis

The data are expressed as mean ± *SEM*. Differences among groups were evaluated using Student's *t*-test of unpaired data, Mann-Whitney Rank Sum Test, or one-way analysis of variance (ANOVA) and Bonferroni's pos*t*-test. All calculations were performed with SPSS 17.0 software (IBM Software, Chicago, IL). Differences with *P* < 0.05 were considered significant.

## SUPPLEMENTARY MATERIALS FIGURE


