# Supplementary material for: MiR-124 aggravates failing hearts by suppressing CD151-facilitated angiogenesis in heart
Source: Oncotarget. 2018 Jan 12;9(18):14382–96. doi: 10.18632/oncotarget.24205 (PMC5865677; doi:10.18632/oncotarget.24205)
Supplement: Supplementary file 1 [file oncotarget-09-14382-s001.pdf]

MiR-124 aggravates failing hearts by suppressing CD151-facilitated angiogenesis in heart

SUPPLEMENTARY MATERIALS

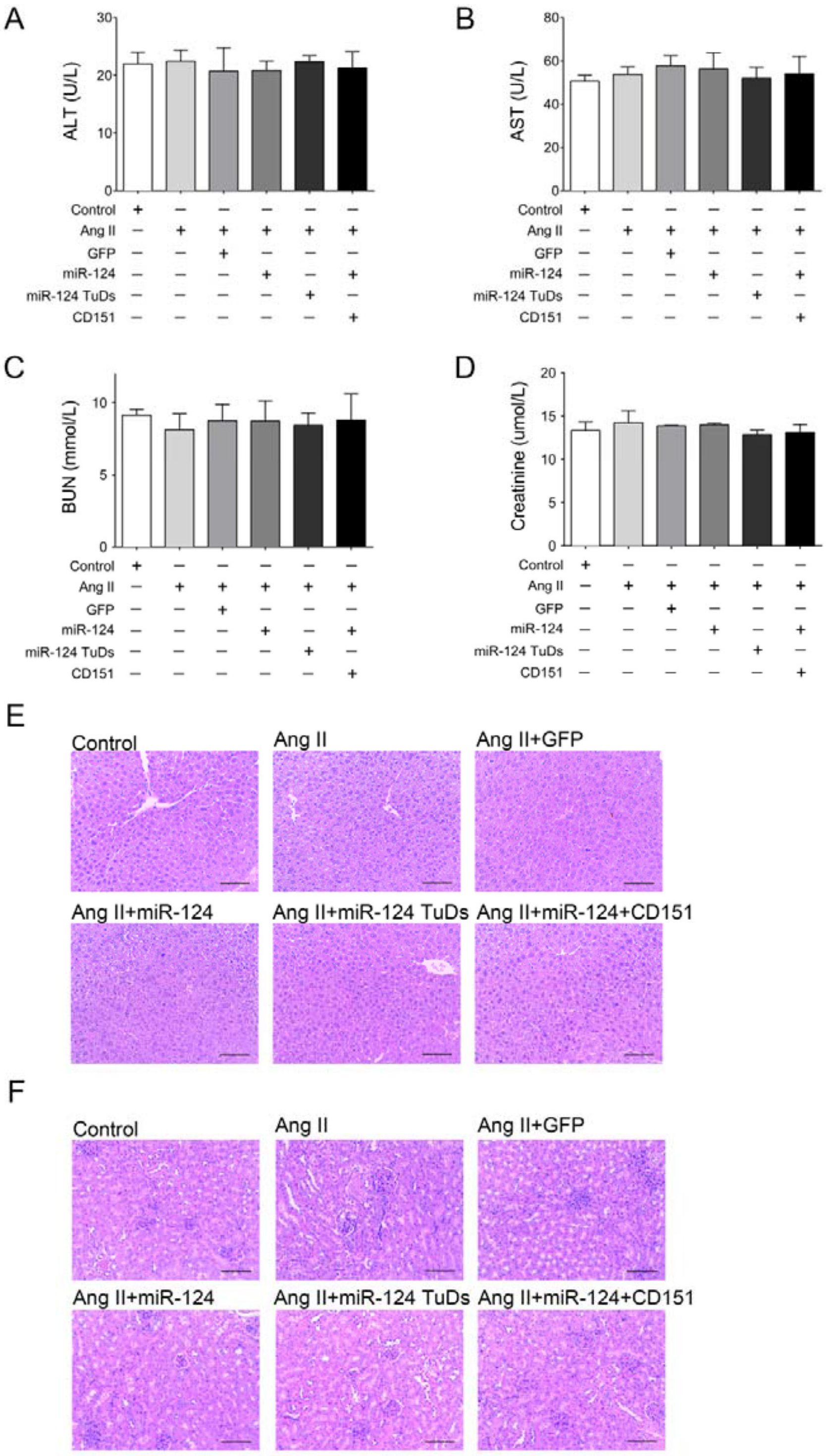

Supplementary Figure 1: Liver and renal function tests and morphologic features. (**A**) Serum ALT, AST (**B**), BUN (**C**) and creatinine (**D**) determined in mice with different treatments. (**E**) Representative images (200X) of H&E staining of livers and kidneys (**F**) from mice that received different treatments. Scale bar, 100 um.
